# Burnout and Psychosocial Risks Among Doctors Working in the Private Sector: The Role of Health and Wellbeing Resources

**DOI:** 10.3390/ijerph22091427

**Published:** 2025-09-12

**Authors:** Kevin Rui-Han Teoh, Oliver Bullock, Marleen Reinke, Gail Kinman, Nicola Cordell, Jo Yarker

**Affiliations:** 1 Birkbeck Business School, Birkbeck, University of London, London WC1E 7HX, UK; marleen.reinke@affinityhealthatwork.com (M.R.); gail.kinman@bbk.ac.uk (G.K.); jo.yarker@affinityhealthatwork.com (J.Y.); 2 Affinity Health at Work, London SW12 9NW, UK; oliver.bullock@affinityhealthatwork.com; 3 Cordell Health, Portsmouth PO6 3TH, UK; nikki@cordellhealth.co.uk

**Keywords:** burnout, private practice, doctors, psychosocial risks, wellbeing resources, occupational health, job demands-resources model, healthcare workforce, dual-sector employment, mental health support

## Abstract

The increasing prevalence of private-sector work among doctors raises questions about its impact on their health and wellbeing. While private practice may offer autonomy and financial benefits, it presents unique psychosocial risks that are less understood. This study investigates the relationship between private-sector work, psychosocial working conditions, and burnout among doctors, and examines whether access to health and wellbeing resources changes these relationships. A cross-sectional survey with 509 doctors from 16 countries working either exclusively or partially in private practice assessed psychosocial risk factors (e.g., work demands, financial pressures, support, job control), and burnout, alongside access to and use of wellbeing resources. Results showed that more time in private practice was associated with higher work and financial demands, bullying, and burnout. Although access to health and wellbeing resources was initially linked to lower burnout, this relationship was not significant when psychosocial risk factors were accounted for. These findings suggest that support mechanisms are often reactive and insufficient in mitigating the impact of systemic demands. The study highlights the need for private healthcare employers to recognise their role in developing healthy work environments, and for organisational-level interventions to address the root causes of poor health and wellbeing among doctors in this sector.

## 1. Introduction

Healthcare systems around the world are changing, and one of the more notable shifts is the growing number of doctors taking on work in the private sector in addition to, or instead of, the public sector. In countries such as Australia, more than 50% of doctors practice privately while in Portugal, 52% work across both sectors [1]. The United Kingdom has observed significant growth in the number of doctors working partially or entirely in the private sector [2]. This trend appears to reflect mounting pressures on public healthcare systems, including funding constraints and long waiting times, alongside a rising demand for private care [3,4]. While working in the private sector, may offer greater autonomy and financial reward; it also presents a set of occupational challenges that have yet to be fully examined.

Burnout is a significant concern within the healthcare workforce, with serious implications for staff wellbeing and the quality of patient care [5]. According to the International Classification of Disease, burnout is a syndrome resulting from work-related stress characterised by emotional exhaustion, depersonalisation, and diminished personal achievement [6]. Evidence consistently links it to negative outcomes, including higher staff turnover, reduced patient satisfaction, and a greater risk of clinical error [7,8,9]. While research increasingly focuses on the role of workplace characteristics as antecedents to doctors’ burnout [7,10], less is known about the impact of doctors working in private practice.

There are good reasons to consider private-sector work as a distinct occupational environment. Whereas public healthcare systems tend to be shaped by policy mandates and clinical priorities, private healthcare is governed by market logic. Doctors in private practice often face different pressures—ranging from financial performance and business sustainability to the management of client relationships [3,11]. These can take the form of revenue targets, customer service expectations, and practice management responsibilities. Other potential hazards for doctors working in private practice include time pressures, scheduling conflicts, administrative and bureaucratic burden, as well as the need to maintain professional standards and manage legal and regulatory risks [12,13]. Not only can such demands increase workload, but they may also create moral tensions that are less visible in public systems [14]. For doctors who are self-employed, working across different organisations can mean that access to organisational support is inconsistent, intensifying the risk of burnout [15].

From a theoretical perspective, the Job Demands–Resources (JD-R) model offers a useful starting point for exploring how these challenges relate to burnout. The model suggests that high job demands—such as work demands, long hours, and time pressure—can lead to burnout, particularly when they are not offset by sufficient job resources like autonomy, peer support, or a sense of control and fairness [16,17]. Although widely applied in occupational health research, the JD-R model within private-sector medicine remains underexplored. This is important given that the relevance and saliency of different aspects of the psychosocial working environment (i.e., job demands and resources) are contextual [18]. For example, recent findings indicate that some resources, including autonomy, may not always be protective. In certain settings, greater control may coincide with heightened responsibility—especially over financial outcomes—and this can exacerbate rather than relieve stress [19].

In response to growing concern about burnout, many healthcare employers have introduced individual-focused wellbeing supports—such as mental health resources, stress management training, or reflective practice spaces [20]. While often well-intentioned, these interventions are not always implemented in ways that address the root causes of stress. Research suggests that such resources tend to be accessed reactively, once symptoms have already emerged, rather than proactively as part of everyday practice [21]. Their effectiveness may also be limited if they are delivered without tackling broader structural or cultural contributors to stress [20,22]. For doctors working independently—especially in private practice—the barriers to accessing support may be even greater, with stigma, cost, and lack of awareness of the available support and limited opportunities to access it all playing a role [19,23].

This intersection between private practice work, psychosocial working environment, and access to wellbeing support raises important questions. Although some research suggested that private-sector doctors may experience higher overall satisfaction [24,25,26], other studies paint a more complex picture. In some high-intensity specialties, such as surgery and urology, burnout may be especially pronounced in private practice settings [27,28]. These patterns point to the need for updated, cross-national data that reflect the current realities of medical practice.

This study contributes to that effort by examining how private-sector employment relates to psychosocial risks and burnout in doctors. Drawing on responses from an international survey of doctors across 16 countries that offer opportunities for private practice work, we explore the prevalence and impact of key factors of the psychosocial working environment salient in the academic literature as predictors of burnout—including workload, financial pressures, and bullying—as well as the availability and use of health and wellbeing resources. We also test whether these health and wellbeing resources have any impact on burnout when considering the impact of the psychosocial working environment. In doing so, this study adds to a more nuanced understanding of occupational wellbeing in contemporary healthcare. More specifically, we aim to address the following three questions:

1. To what extent is private-sector work associated with the psychosocial working environment and burnout among doctors?
2. How does access to, and use of, wellbeing resources relate to these outcomes?
3. Does the relationship between these resources and burnout change depending on the psychosocial working environment of doctors?

## 2. Methodology

### 2.1. Study Design and Sample

This cross-sectional online survey, administered through Qualtrics, ran from June to August 2024. The survey was part of a wider mixed-methods study focusing on the working experience and wellbeing of doctors and dentists working in public and private practice globally. Participants were recruited using a convenience sampling approach via social media and emails circulated via the networks of the MPS Foundation, part of the Medical Protection Society, a member-owned, not-for-profit protection organisation for doctors, dentists and healthcare professionals. Participants in this study had to be currently practicing or recently retired doctors working either wholly privately or in a dual role (private and public).

### 2.2. Measures

Participants provided sociodemographic information (age, tenure, employment status) as control variables, along with validated measures of their working conditions and burnout. Further questions assessed the availability of health and wellbeing support within organisations. The survey was pilot tested for face validity with a steering group consisting of researchers, doctors, practitioners, and professional body representatives. Participants were also asked to estimate the proportion of their time that they worked in private practice (from 0 to 100%).

### 2.3. Psychosocial Working Environment

We measured *workload* using four items from the work demands subscale of UK Health and Safety Executive’s (HSE) management standards framework (*α* = 0.87). For *financial demands* we used a single item “I experience pressure related to financial targets at work” from the Veterinarian Stressors Inventory [29] but adapted for the context of this study. From the Copenhagen Psychosocial Questionnaire [30], we used one item each to measure *bullying* (i.e., “I have been subjected to bullying at work in the last 6 months”) and *collegial support* was measured with one item adapted from the Copenhagen Psychosocial Questionnaire (“I have good relationships with co-workers/others in my professional network”). Three items from Breaugh’s Work Autonomy Scale [31] measured *work control* (*α* = 0.81). All participants were asked to focus on their work in private practice and to rate the frequency of their experiences on a 5-point Likert scale from never (1) to always (5) for all items—in line with the format of the HSE management standards measure [32].

### 2.4. Burnout and Wellbeing Support

We used a single item measure of burnout: “I feel burned out from work” scored on a 7-point Likert scale (1 = “never”, 7 = “every day”) [33]. Three items measured health and wellbeing support within organisations, with the examples of counselling, stress management training, and mental health awareness training provided. Participants were asked to think about the resources available to them in private practice, and then whether they (i) have access to; (ii) have ever accessed; and (iii) would access these resources in times of need. Responses were coded as yes, no, or maybe.

### 2.5. Ethical Considerations

The study was conducted according to the guidelines of the Declaration of Helsinki, and approved by the Ethics Committee of Birkbeck, University of London (Reference: clxj1459s001xl07lkhlx42bk) on 13 June 2024. Informed consent was obtained from all subjects involved in the study. Participants were presented with the study information and provided consent via a tick box prior to completing the online survey. No directly identifiable information (e.g., IP addresses, email addresses) was collected.

### 2.6. Data Analysis

We carried out all analyses conducted using SPSS version 29. Prior to analysis, data were screened for missing values and outliers and then tested for normality and linearity. Cases with incomplete responses beyond demographic items were excluded, resulting in a final sample of 509 participants.

Descriptive statistics and Pearson’s correlation coefficients were computed to examine relationships among key study variables, including time spent in private practice, psychosocial working environment, and burnout. We then used independent samples *t*-tests to compare psychosocial working environment and burnout scores between groups based on access to, use of, and willingness to use health and wellbeing resources.

To test the predictors of burnout, we ran a hierarchical multiple regression analysis with burnout as the dependent variable. This method allowed for testing the incremental contribution of different blocks of variables in predicting burnout, the relative influence of control variables, wellbeing resources, and psychosocial risks in a theoretically informed sequence. It is particularly suited to evaluating models based on the JD-R framework, where the goal is to assess whether resources buffer the effects of demands. Variables were selected based on their relevance to the JD-R model and prior empirical evidence linking them to burnout in healthcare settings [34]. In Step 1, control variables (age, tenure, and percentage of time spent in private practice) were entered to account for baseline differences. In Step 2, access to and use of health and wellbeing resources were added to test their direct effects. In Step 3, psychosocial working environment variables (work demands, financial demands, bullying, collegial support, and control) were included to assess their incremental predictive value and to examine whether the effects of health and wellbeing resources remained significant after accounting for psychosocial working factors.

## 3. Results

A total of 527 participants answered this survey, with 509 usable responses (*n* = 509). Most doctors were self-employed (64.9%), with 21.3% employed by one organisation and 13.9% by multiple organisations. The most common age was between 45 and 54 years (35.2%) and 55–64 years (31.2%), followed by those aged 35–44 years (15.7%) and 65–74 years (12.8%). Most participants had worked in private practice for more than 10 years (65.1%) followed by 1–4 years (15.6%) and 5–10 years (15%). Participants responded from 16 countries (see Table A1 for full participant breakdown), with the largest proportion from South Africa (33.7%), United Kingdom (32.2%), Ireland (16.5%), Singapore (4.0%), Hong Kong (3.6%), and Malaysia (3.6%). All participants worked in private practice with, on average, participants spending 71.82% of their time working in private practice. This ranged from 8.2% of participants who spent less than 10% of their time in private practice to 53.2% who worked exclusively in private practice.

Table 1 presents the descriptive details and correlation matrix for the study variables. The proportion of time in spent in private practice was positively correlated with work (*r* = 0.32, *p* < 0.001 and financial (*r* = 0.32, *p* < 0.001) demands, as well as bullying (*r* = 0.13, *p* < 0.01) and burnout (*r* = 0.19, *p* < 0.001). Colleague support had no relationship with time spent in private practice. All other relationships were observed as anticipated.

## 4. Access and Use of Health and Wellbeing Resources in Private Practice

When asked about health and wellbeing resources in private practice, 38.6% reported they had access to them, 21.9% had utilised them, and 61% indicated they would use them in times of need (Table 2).

Independent samples *t*-test were conducted to examine whether there were differences on the measures of the work environment and burnout between those who responded “yes” versus “no” on the questions in Table 2. Means and inferential statistics for these results are presented in Table A2.

Doctors who reported that they had access to health and wellbeing resources in private practice had spent a higher proportion of their time in private practice (*M* = 81.79, *SD* = 31.13) than those who said they did not (*M* = 71.02, *SD* = 36.12; *t*(351) = −3.00, *p* < 0.01). They also reported lower work demands scores (*M* = 2.24, *SD* = 1.23 vs. *M* = 2.67, *SD* = 1.08; *t*(359) = 1.76, *p* < 0.05) but more collegial support (*M* = 4.34, *SD* = 0.64 vs. *M* = 4.18, *SD* = 0.84; *t*(358) = −2.01, *p* < 0.05) than those who said they did not.

Comparisons on participants who have used the health and wellbeing resources (Table 3) show that those who did use the resources to report higher levels of work demands (*t*(488) = −3.63, *p* < 0.001), financial demands (*t*(488) = −3.91, *p* < 0.001), bullying (*t*(487) = −2.43, *p* < 0.05), and burnout (*t*(488) = −2.27, *p* < 0.05) than those that did not use these resources. Those who had accessed resources also spent a larger proportion of their time in private practice (*t*(478) = −2.25, *p* < 0.05) than those who did not.

When asked whether they would access health and wellbeing resources in times of need, those who said yes spent a much larger proportion of their time in private practice (*M* = 75.59, *SD* = 58.83) than those who said they did not (*M* = 58.53, *SD* = 38.01; *t*(370) = −3.68, *p* < 0.001). However, there were no differences on any of the measures (Table 3).

## 5. Predictors of Burnout

A hierarchical regression was conducted to examine predictors of burnout (Table 3). In Step 1, control variables (age, tenure, and percentage of time spent in private practice) accounted for 9.8% of the variance in burnout: R^2^ = 0.098, F(3, 480) = 17.39, *p* < 0.001. Age was negatively associated with burnout (β = −0.30, *p* < 0.001), while tenure (β = 0.15, *p* = 0.005) and time in private practice (β = 0.15, *p* < 0.001) were positively associated with burnout.

In Step 2, access to and use of health and wellbeing resources were added. This model explained an additional 1.7% of the variance: ΔR^2^ = 0.017, F change(2, 478) = 6.16, *p* < 0.01. Access to resources significantly predicted lower burnout (β = −0.13, *p* < 0.01).

In Step 3, psychosocial working conditions were added (work demands, financial demands, control, bullying, and collegial support). The model significantly improved: R^2^ = 0.426, ΔR^2^ = 0.311, F change(5, 473) = 35.13, *p* < 0.001. Higher work demands were the strongest predictor of burnout (β = 0.51, *p* < 0.001), while meeting financial demands was also positively associated with burnout (β = 0.089, *p* < 0.05). Access to and use of wellbeing resources were no longer significant predictors of burnout once when psychosocial working factors were accounted for. While wellbeing resources may offer some protective effect against burnout in isolation, this effect disappears when work-related factors are considered, suggesting that the influence of the psychosocial work environment outweighs that of individual access to support.

## 6. Discussion

This study explored the psychosocial risks and burnout experienced by doctors working in private practice, either wholly or partially. Our findings reveal that doctors who spend more time in private practice report higher levels of work demands, financial pressures, emotional demands, and bullying—factors that are consistently associated with elevated burnout. While access to health and wellbeing resources was associated with lower burnout in initial models, this protective effect disappeared once the psychosocial working environment was accounted for. These results suggest that, while individual-focused resources may offer some benefit, they alone are likely insufficient in the face of persistent and systemic workplace demands.

The results align with the JD-R model which postulates that burnout arises when job demands exceed available resources [7,16]. Our findings also challenge the assumption that access to resources alone can buffer the effects of high demands. Doctors who had accessed health and wellbeing resources reported higher levels of work and financial demands, as well as bullying—suggesting that engagement with support mechanisms is primarily reactive rather than preventive. This pattern may reflect a broader issue in healthcare where support systems are often more reliant on individual initiatives [35,36]. It also is congruent with the extant literature highlighting the psychosocial working environment as a key antecedent to doctors’ health and wellbeing over and above individual factors [20,37].

Our findings are also congruent with a large body of evidence demonstrating that work demands are a key predictor of burnout among doctors [5,7], although the role of financial demands as a predictor has, to date, received limited attention. In private practice, doctors may face additional pressures related to financial performance [3] that not only relate to those self-employed but may face demands around fee generation that can be time consuming and stressful. In turn, this financial demand may amplify the risk posed by traditional work demands. Similarly, the potential of collegial support and control as resources that buffer may lack salience amongst doctors who take on private-sector work, especially if they work across several organisations. As, on average, participants still spent a quarter of time working for the public sector and with nearly 65% reporting being self-employed, the multiple roles undertaken and lack of organisational embeddedness may limit doctors’ ability to exert control over their working environment or to develop supportive collegial relationships [38,39]. This lack of institutional support for self-employed doctors further compounds this issue, as they may not have access to structured wellbeing programmes or occupational health services, or that the stigma associated with help-seeking in medicine is compounded when doctors do not identify with the organisations they work with or where they work on a self-employed basis.

From a theoretical perspective, this study contributes to the refinement of the JD-R model by highlighting the limitations of individual-level resources in high-demand, low-support environments such as private practice. The finding that access to wellbeing resources loses its protective effect when psychosocial risks are accounted for suggests that the buffering role of resources may be conditional on the broader organisational context [40]. This is particularly the case in private practice, where organisational context may be fragmented or absent. The lack of a centralised employer or governing body in many private settings complicates the implementation of systemic interventions, making it unclear who is responsible for fostering healthy work environments. This ambiguity challenges traditional applications of the JD-R model, which often assume the presence of a cohesive organisational structure. Moreover, the possibility that certain resources—such as job control—were not associated with burnout challenges the assumption that all resources are inherently protective. It may be that in these contexts, participants did not see job control as a resource or that it may have a ceiling effect. Future research should also explore models of accountability and governance in private healthcare settings. Understanding who holds responsibility for clinician wellbeing (e.g., practice owners, professional bodies, or regulators) is essential for designing effective interventions. Comparative studies between public and private sectors could further illuminate how structural differences shape both risk exposure and the feasibility of change. Finally, there needs to be acknowledgement of the broader systematic factors that impact the experience of job demands and resources. Here, models such as IGLOO [41] allow the exploration of factors beyond the individual, recognising the importance of the group, leader, organisation, and overarching context. Alternatively, the literature around psychosocial safety climate posits that shared perceptions of organisational policies, practices, and procedures for protecting psychological health and safety are antecedents to job demands and resources [42]. Collectively, these insights call for a more nuanced application of the JD-R model that accounts for sector-specific dynamics and the potential for non-linear or context-dependent effects [18].

## 7. Implications for Practice

Reflecting on the study findings, there are several implications for practice to be considered. Crucially, they reinforce the notion that burnout is not merely a result of an individual struggling to cope, but an outcome of wider organisational and systematic conditions [5,10], with considerations for private healthcare employers, public healthcare employers, and individual doctors.

Private healthcare employers must recognise their legal and ethical responsibility to provide psychologically safe work environments [20]. However, the unique context of private practice complicates this responsibility. Unlike public healthcare systems, which are embedded within larger institutional frameworks, private practices often operate independently, with varying degrees of organisational structure and oversight [43]. This raises an important and underexplored question: Who is accountable for improving the culture, psychological safety, and workload conditions in private practices? In many cases, the responsibility may fall to individual practice owners or small management teams who may lack the resources, training, or incentives to implement systemic change. The study highlights the need to better understand and manage the sources of psychosocial demands in private practice, including financial targets, workload expectations, and interpersonal dynamics. Employers should be equipped with the knowledge and tools to assess psychosocial risks and implement organisational-level interventions [20]. This includes reviewing workload and financial expectations, improving access to support for self-employed and dual-role doctors, and promoting a culture of openness around mental health to reduce stigma and encourage early help-seeking. Training managers and practice owners in psychosocial risk assessment and supportive leadership is also essential [44,45]. Furthermore, the development of sector-wide standards for wellbeing provision in private healthcare, aligned with occupational health best practices, could help ensure more consistent and equitable support for clinicians.

Although public healthcare employers are not directly responsible for stressors experienced in private practice, they should be aware of the cumulative demands placed on staff who work across sectors. Dual-sector doctors may carry additional burdens that affect their performance and wellbeing in public roles [46]. There is an opportunity for public and private providers to collaborate in offering shared access to specialist support services, ensuring that dual-role doctors are not left unsupported. This could link to the support services provided by professional bodies and regulators to individual doctors, but also crucially, the role of these stakeholders in overseeing training, work processes and regulations that impact the demands and resources that doctors carrying out private practice experience [47,48]. Therefore, coordinated messaging and paired wellbeing initiatives could help bridge this gap and promote a more integrated approach to doctors’ health and wellbeing.

While the onus is on stakeholders to facilitate a better psychosocial working environment, there are some actions that can be taken by individuals. For doctors working in private practice, particularly those balancing dual roles, the results underscore the importance of proactively managing their working environments. Where institutional support may be limited or absent, doctors may need to take additional steps to access wellbeing resources, build peer networks, and advocate for safer working conditions. Here, job crafting, which refers to the self-initiated changes a worker does to manage their demands and build resources [49], may have a role to play, especially as it has been found to be effective improving the health and wellbeing of healthcare professionals [50]. Developing skills to recognise psychosocial hazards and engage in constructive dialogue with employers could empower doctors to take preventive action and reduce the risk of burnout.

## 8. Strengths and Limitations

This study offers several strengths. It draws on a large, international sample of doctors from 16 countries, providing a rare cross-national perspective on private-sector working conditions. It also combines validated measures of psychosocial risks and burnout with novel data on access to wellbeing resources—an under-researched area in occupational health.

Despite these strengths, there are several limitations that need to be acknowledged. First, as covered earlier, the cross-sectional study design does not allow for any causal inferences, with the addition of self-reported measures meaning that the results are potentially affected by common method variance which may inflate observed relationships due to shared measurement context [51]. To mitigate this, we used validated instruments, varied response formats, and ensured anonymity to reduce social desirability bias. Nonetheless, future studies should consider using multi-source data or temporal separation of measures to further reduce this risk. Second, we did not distinguish between doctors who worked solely in private practice and those who worked in both private and public sectors, and it may be that those who have to juggle both sectors have a different experience to those who can focus on only the private sector. There may also be systematic difference between those who access wellbeing resources to those who do not (or are aware of them). Linked to this, the spread of participants’ countries represents a diverse spread of healthcare systems and demands, and it is vital to recognise that there may be significant variations in the work experiences across countries [52]. These differences in contextual factors (e.g., financial reimbursement models, patient care expectations, or health system structures) are all potential confounders that should be accounted for. Third, the use of a single-item burnout measure, while validated, may not capture the full complexity of the construct [33]. Fourth, while we grounded the job demands and resources in this study within the extant literature, other constructs—such as patient and administrative demands [3,4], may be more congruent to this study sample. Similarly, we focus on perceived demands and resources when variables about the actual work environment (e.g., average patient panel size and flexibility in scheduling) would provide a different perspective on burnout drivers [53]. Finally, there is a clear gap for qualitative research to unpack the nuances of doctors’ experiences in greater depth—particularly in relation to accessing support [23].

## 9. Conclusions

As healthcare systems evolve and the demand for private healthcare grows, increasing numbers of doctors are likely to work in private or dual-sector roles. This study demonstrates that private practice is not immune to the psychosocial risks that drive burnout. In fact, doctors in these settings face significant challenges—including high work demands, financial pressures, and bullying—that can undermine their wellbeing. While access to support services is important, it alone is not sufficient. Addressing the root causes of stress within the work environment must be a priority. Raising awareness of these issues within the private healthcare sector is essential to protect the health of clinicians and the safety of patients.

## Figures and Tables

**Table 1:** Descriptive Statistics, Reliabilities, and Intercorrelations Among Study Variables.

Measure	M	SD	1	2	3	4	5	6
1. Proportion of time in private practice	71.82	36.22	-					
2. Work demands	2.49	0.99	0.32 **	-				
3. Financial demands	2.19	1.23	0.32 **	0.53 **	–			
4. Bullying	1.71	0.95	0.13 **	0.42 **	0.30 **	–		
5. Collegial support	4.26	0.75	–0.02	–0.13 **	–0.10 *	–0.31 **	–	
6. Control	3.79	0.92	–0.17 **	–0.43 **	–0.24 **	–0.40 **	0.23 **	-
7. Burnout	3.05	1.68	0.19 **	0.61 **	0.40 **	0.36 **	–0.19 **	–0.35 **

Note. * *p* < 0.05, ** *p* < 0.01.

**Table 2:** Frequency of Responses on Access and Use of Health and Wellbeing Resources.

**Wellbeing Resources in Private practice**	**Yes**	**No**	**Not sure**
Have access to resources	193 (38.6%)	168 (33.6%)	139 (27.8%)
Have ever accessed resources	110 (21.9%)	380 (75.7%)	12 (2.4%)
Would access resources in time of need	306 (61%)	75 (14.9%)	121 (24.1%)

**Table 3:** Hierarchical Regression Predicting Burnout.

Predictor	Step 1 B (SE)	β	Step 2 B (SE)	β	Step 3 B (SE)	β
Constant	4.48 (0.62) ***	-	4.72 (0.65) ***	-	2.85 (0.69) ***	-
Age	−0.49 (0.08)	−0.30 ***	−0.49 (0.08)	−0.30 ***	−0.18 (0.07)	−0.11 *
Tenure	0.27 (0.10)	0.15 **	0.27 (0.10)	0.14 **	0.03 (0.08)	0.02
% Private practice	0.01 (0.01)	0.15 ***	0.01 (0.01)	0.16 ***	−0.01 (0.01)	−0.03
Have access to resources	-	-	−0.26 (0.09)	−0.13 **	−0.02 (0.08)	−0.01
Have ever accessed resources	-	-	0.18 (0.09)	0.09	−0.05 (0.08)	−0.03
Work demands	-	-	-	-	0.88 (0.08)	0.51 ***
Financial demands	-	-	-	-	0.13 (0.06)	0.09 *
Bullying	-	-	-	-	0.10 (0.08)	0.06
Collegial support	-	-	-	-	−0.17 (0.09)	−0.07
Control	-	-	-	-	−0.10 (0.08)	−0.05
R^2^	0.10	-	0.12	-	0.43	-
ΔR^2^	-	-	0.017 **	-	0.311 ***	-
F for change	-	-	6.16 **	-	35.13 ***	-

Note. B = unstandardized regression coefficient; SE = standard error; β = standardised coefficient. * *p* < 0.05, ** *p* < 0.01 and *** *p* < 0.001.

## Data Availability

Anonymised data with limited demographic details presented in this study are available on request from the corresponding author.

## Appendix A

**Table A1:** Demographic Details of Participants.

Demographic	Response	N (%)
Employment Status	Self-employed	342 (64.9%)
	Single employer	112 (21.3%)
	Multiple employers	73 (13.9%)
Tenure in Private Practice	Less than 1 year	21 (4.0%)
	1–4 years	82 (15.6%)
	5–10 years	79 (15.0%)
	More than 10 years	343 (65.1%)
	Prefer not to say	2 (0.4%)
Age	25–34 years	13 (2.5%)
	35–44 years	82 (15.7%)
	45–54 years	184 (35.2%)
	55–64 years	163 (31.2%)
	65–74 years	67 (12.8%)
	75+ years	13 (2.5%)
Country	South Africa	178 (33.7%)
	United Kingdom	170 (32.2%)
	Ireland	87 (16.5%)
	Singapore	21 (4.0%)
	Hong Kong	19 (3.6%)
	Malaysia	19 (3.6%)
	Trinidad and Tobago	9 (1.7%)
	Namibia	8 (1.5%)
	Australia, Barbados, Jamaica, New Zealand, Papua New Guinea	2 (0.4%)
	Colombia, Netherlands, Saint Kitts and Nevis	1 (0.2%)

**Table A2:** Means and Independent *t*-tests Comparing the Study Variables for Those Who Have Versus Whose Have Not Accessed Health and Wellbeing Resources.

		Have Access to Resources			Have Ever Accessed Resources			Would Access Resources in Time of Need		
		N	Mean (SD)	t(df) = Value	N	Mean (SD)	t(df) = Value	N	Mean (SD)	t(df) = Value
Time spent in private practice	Yes	190	81.79 (31.13)	*t*(351) = −3.01 **	110	78.74 (33.96)	*t*(478) = −2.25 *	300	75.59 (34.70)	*t*(370) = −3.68 ***
	No	163	71.02 (36.12)		370	69.91 (36.69)		72	58.53 (38.01)	
Work demands	Yes	193	2.48 (0.69)	*t*(359) = 1.76 *	110	2.80 (0.76)	*t*(488) = −3.63 ***	306	2.55 (1.00)	*t*(379) = −1.30
	No	168	2.67 (0.76)		380	2.41 (0.70)		75	2.38 (1.10)	
Financial demands	Yes	193	2.24 (0.76)	*t*(359) = 0.73	110	2.59 (0.79)	*t*(488) = −3.91 ***	306	2.26 (1.22)	*t*(379) = −1.24
	No	168	2.34 (0.74)		380	2.08 (0.72)		75	2.07 (1.32)	
Bullying	Yes	193	1.74 (0.68)	*t*(358) = 0.97	110	1.89 (0.73)	*t*(487) = −2.43 *	306	1.71 (0.89)	*t*(378) = 0.52
	No	167	1.84 (0.71)		379	1.64 (0.65)		74	1.77 (1.20)	
Collegial support	Yes	193	4.34 (0.65)	*t*(358) = −2.01 *	110	4.27 (0.69)	*t*(487) = 0.05	306	4.31 (0.73)	*t*(378) = −0.98
	No	167	4.18 (0.69)		379	4.28 (0.65)		74	4.22 (0.80)	
Control	Yes	193	3.75 (0.72)	*t*(359) = −0.53	110	3.69 (0.77)	*t*(488) = 1.37	306	3.77 (0.94)	*t*(379) = 1.55
	No	168	3.70 (0.77)		380	3.83 (0.74)		75	3.96 (0.91)	
Burnout	Yes	193	3.04 (1.05)	*t*(359) = 1.36	110	3.38 (1.07)	*t*(488) = −2.27 *	306	3.11 (1.71)	*t*(379) = −0.32
	No	168	3.28 (1.03)		380	2.97 (1.01)		75	3.04 (1.85)	

Note. * *p* < 0.05, ** *p* < 0.01 and *** *p* < 0.001.
